# An Inverse Neural Controller Based on the Applicability Domain of RBF Network Models

**DOI:** 10.3390/s18010315

**Published:** 2018-01-22

**Authors:** Alex Alexandridis, Marios Stogiannos, Nikolaos Papaioannou, Elias Zois, Haralambos Sarimveis

**Affiliations:** 1 Department of Electronic Engineering, Technological Educational Institute of Athens, Agiou Spiridonos, 12243 Aigaleo, Greece; mstogia@teiath.gr (M.S.); nck.papaioannou@gmail.com (N.P.); ezois@teiath.gr (E.Z.); 2 School of Chemical Engineering, National Technical University of Athens, Iroon Polytechneiou 9, Zografou, 15780 Athens, Greece; hsarimv@central.ntua.gr

**Keywords:** applicability domain, data fusion, intelligent control, neural networks, radial basis function

## Abstract

This paper presents a novel methodology of generic nature for controlling nonlinear systems, using inverse radial basis function neural network models, which may combine diverse data originating from various sources. The algorithm starts by applying the particle swarm optimization-based non-symmetric variant of the fuzzy means (PSO-NSFM) algorithm so that an approximation of the inverse system dynamics is obtained. PSO-NSFM offers models of high accuracy combined with small network structures. Next, the applicability domain concept is suitably tailored and embedded into the proposed control structure in order to ensure that extrapolation is avoided in the controller predictions. Finally, an error correction term, estimating the error produced by the unmodeled dynamics and/or unmeasured external disturbances, is included to the control scheme to increase robustness. The resulting controller guarantees bounded input-bounded state (BIBS) stability for the closed loop system when the open loop system is BIBS stable. The proposed methodology is evaluated on two different control problems, namely, the control of an experimental armature-controlled direct current (DC) motor and the stabilization of a highly nonlinear simulated inverted pendulum. For each one of these problems, appropriate case studies are tested, in which a conventional neural controller employing inverse models and a PID controller are also applied. The results reveal the ability of the proposed control scheme to handle and manipulate diverse data through a data fusion approach and illustrate the superiority of the method in terms of faster and less oscillatory responses.

## 1. Introduction

Artificial neural networks (NNs) possess several properties that make them particularly suitable for modeling and control applications in nonlinear systems engineering. The most important among these is their ability to learn complex and nonlinear relationships without explicit knowledge of the first-principle equations describing the system, but based solely on input-output data from it. This basic feature is complemented by other desirable properties such as universal approximator capabilities, tolerance to faults and uncertainties, massive parallel processing of information, and the ability to perform data fusion, i.e., to handle and merge data from multiple sources.

The potential of integrating NN technologies in control systems to deal effectively with challenging nonlinear control problems was identified from the early 1990s. The seminal survey on the use of neural networks for control systems [1] was followed by a number of scientific books, specializing in the combination of NNs and control engineering [2,3]. More than two decades later, besides being the objective of numerous theoretical studies, the utilization of NNs in control systems has started to penetrate in the industrial market [4,5]. Yet, there are still many open issues that need to be resolved, pertaining to optimizing the performance of NN-based control systems, as well as increasing their reliability.

NNs have been thoroughly exploited in different variants of the backstepping control technique [6]. Backstepping assumes that a dynamic system model is available, and has been shown to work successfully even in the presence of uncertainty in the model parameters. In this context, NNs are used to estimate unknown nonlinear functions in the backstepping design, so that the linear-in-the-parameters assumption can be avoided. On the other hand, there are two basic data-driven approaches for formulating NN-based control strategies, which do not assume any previous knowledge of the system dynamics: indirect design, where an NN is trained as a dynamic system model, predicting the states and/or the output; and direct design, where the NN approximates the inverse system dynamics and acts as a controller.

As far as indirect design is concerned, the NN model cannot by itself be used to control the plant, as it is trained only to identify the unknown system and predict the result of candidate control actions. For this reason, indirect design techniques couple the NN model with appropriately designed control laws. A popular approach within this framework is the model predictive control (MPC) method, where the NN model is inversed through an optimization procedure, in order to estimate the optimal sequence of control actions, which brings the system to the desired conditions. In this context, an MPC controller based on linearized NN models for reducing the computational burden is developed in [7]. An NN approach to robust MPC for constrained nonlinear systems with unmodeled dynamics is introduced in [8]. A nonlinear NN-based MPC controller, for use in processes with an integrating response exhibiting long dead time, is designed and successfully applied to the temperature control of a semi-batch reactor in [9]. The calcination process within the production of high-octane engine fuels is shown to be successfully under control of a NN-based MPC integrated system, and capable of significantly lowering plant costs in [10]. Further progress on neural-based MPC is catalyzed by recent developments in: (a) NN training algorithms, which help to increase the predictive model accuracy and consequently the controller performance [11]; and (b) nonlinear search methods, which enable the incorporation of multi-objective, large-scale optimization problems in MPC [12,13]. Theoretic developments on neural-based MPC were accompanied by several successful real world applications [14,15].

Despite their advantages, indirect design methodologies based on the MPC concept share an important drawback: the optimization problem, which is nonlinear in nature, is formulated for every discrete time instant [16], and needs to be solved online before the next sample is collected from the system. This limitation prevents the application of such methods to systems with inherently fast dynamics. The aforementioned computational issues are efficiently tackled by the direct design approach, in which the produced NN acts as the controller, i.e., at each discrete time step the NN determines the values of the manipulated variables as functions of the current state, or based on past information on input-output variables. Following this approach, the NN is trained to perform as the inverse response of the plant [17], cancelling the system dynamics and making it track the reference input. In [18], a direct adaptive neural speed tracking control technique is presented for permanent magnet synchronous motor drive systems, using NNs to approximate the desired control signals. The discrete direct neural control for flight path angle and velocity of a generic hypersonic flight vehicle (HFV) is investigated in [19]. In [20], a scheme using an NN model for identification and an inverse NN controller is designed for controlling nonlinear dynamic systems, while reducing the network training time. A direct design framework using two learning modules is introduced in [21].

The efficiency of direct design methodologies stems from the fact that their implementation is rather straightforward, as it only requires evaluating a nonlinear function at every time instant, contrary to indirect MPC methods that mandate the solution of a nonlinear optimization problem. On the other hand, direct design-based controllers are usually inferior in terms of optimality and robustness, compared to their indirect design counterparts. In order to boost the performance of direct design techniques, careful design is needed in such aspects as the selection of training data, the NN type, and the training methodology used.

Radial basis function (RBF) networks [22,23] form a special type of NNs with important advantages, including (a) better approximation capabilities when performing interpolation, i.e., providing predictions in-between the training data points; (b) simpler network structures comprising a single hidden layer and a linear connection between the hidden and output layers; and (c) faster learning algorithms, usually split into two stages. Not surprisingly, RBF networks have been used extensively in both indirect and direct controller design approaches [4,11,12,24,25,26,27]. Their main disadvantage is that RBF networks are particularly prone to poor extrapolation, i.e., they fail to provide predictions in areas of the input state-space that lack sufficient training data coverage. Though the universal approximator property guarantees [28] the theoretical existence of an RBF neural network that could approximate any continuous function to an arbitrary accuracy, it does not take into account the availability of training data, which in real world applications may be rather limited. It should be noted that the negative effects of extrapolation are not restricted to RBF networks; other types of NNs have also been shown to perform rather poorly when asked to extrapolate [29]. Unfortunately, it is widely acknowledged that in the general case, the prediction of any NN model as a result of extrapolation cannot be considered reliable. Obviously, the inability of NN models to extrapolate can gravely affect any neural controller, regardless of the type of design being direct or indirect; a poor prediction due to extrapolation could impair the controller performance or even lead to instability. A second issue, more common to direct neural controllers, is the poor ability to take into account model uncertainties introduced by the initial training dataset and unmeasured external disturbances, which usually result in steady offsets [30]. In a recent publication [31], offset-free direct neural control of a chemical reactor exhibiting multiple steady states was achieved by including a mechanism for augmenting the state vector with an additional state that estimates the error due to unmodeled inverse dynamics and/or unmeasured external disturbances.

In the present work, a new direct design method is presented for building neural controllers of generic nature, based on RBF networks. The main contributions of this work are: (a) The proposed methodology uses the particle swarm optimization-based, non-symmetric implementation of the fuzzy means algorithm (PSO-NSFM), which offers increased accuracy and smaller network structures [32] compared to other existing methods. The objective is to accurately capture the inverse system dynamics using data that may originate from multiple sources and are collected during the normal operation of the plant; (b) It is shown that an appropriate choice of basis function guarantees bounded input-bounded state (BIBS) stability for the closed loop, when the open loop is BIBS stable; (c) The resulting model is used in conjunction with a concept known as applicability domain (AD) [33], to ensure that no extrapolation occurs while obtaining the predictions produced by the inverse neural controller; (d) The method incorporates an error correction term, increasing the proposed control scheme’s robustness. Application to two different control problems demonstrates the advantages and the generic nature of the proposed approach.

The rest of the paper is formed as follows: The next section gives a brief presentation of RBF NNs and the PSO-NSFM algorithm. Section 3 is dedicated to the presentation of the proposed controller design method, including a discussion on RBF-based inverse controllers and BIBS stability, the incorporation of AD criterion, and the robustifying error correction term. Section 4 presents the application of the controller in two different control problems, including a comparison with different approaches. Finally, conclusions are drawn in the last section.

## 2. RBF Networks

The input layer of a typical RBF network distributes the N input variables data to the L kernels of the network’s hidden layer. Each kernel node is assigned to a center vector, which is of equal dimensionality to the input space. Thus, a nonlinear transformation is performed by the hidden layer, so as to map the input state-space onto a new space with higher dimensionality. The activity $\mu_{l}\left(u\left(k\right)\right)$ of the lth kernel is given by the Euclidean distance between the kth input vector and the kernel center:

(1) $$\mu_{l}\left(u\left(k\right)\right)=\left\|u\left(k\right)-{\hat{u}}_{l}\right\|=\sqrt{\sum_{i=1}^{N}{\left(u_{i}\left(k\right)-{\hat{u}}_{i,l}\right)}^{2}},\;k=1,\;...,\;K$$

in which K is the number of training data, $u^{T}\left(k\right)=\left[u_{1}\left(k\right),u_{2}\left(k\right),...,u_{N}\left(k\right)\right]$ is the input data vector, and ${\hat{u}}_{l}^{T}=\left[{\hat{u}}_{1,l},{\hat{u}}_{2,l},...,{\hat{u}}_{N,l}\right]$ are the center coordinates of the lth kernel.

The activation function for each node is a function with radial symmetry. In this work, the Gaussian function is employed:

(2) $$g\left(\mu_{l}\right)=\exp\left(-\frac{\mu_{l}{}^{2}}{\sigma_{l}{}^{2}}\right)$$

in which $\sigma_{l}$ are the widths of the Gaussians. The latter can be calculated using the *p*-nearest neighbor technique, which selects the width of each basis function $\sigma_{l}$ as the root-mean squared distance to its *p*-nearest neighbors, using the following equation:

(3) $$\sigma_{l}=\sqrt{\frac{1}{p}\sum_{k=1}^{p}{\left\|{\hat{u}}_{k}-{\hat{u}}_{l}\right\|}^{2}},l=1,...,L$$

in which ${\hat{u}}_{k}$ are the *p*-nearest centers to kernel center ${\hat{u}}_{l}$.

**Figure 1:**
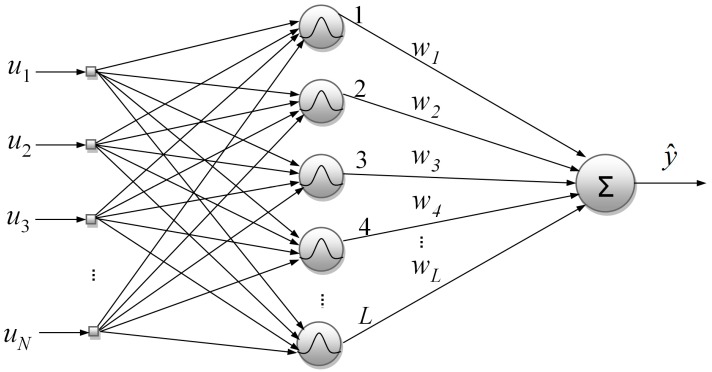
Typical structure of an RBF network with Gaussian basis functions.

Finally, a linear combination of the hidden layer kernel responses produces the RBF network output $\hat{y}\left(k\right)$:

(4) $$\hat{y}\left(k\right)=z\left(k\right)\cdot w=\sum_{l=1}^{L}w_{l}g\left(\mu_{l}\left(u\left(k\right)\right)\right)$$

in which z(k) are the hidden node responses and w is a vector containing the synaptic weights. Figure 1 depicts a typical RBF network with Gaussian basis functions.

Having obtained the RBF kernel centers, linear regression of the hidden layer node outputs to the target values is typically used to calculate the synaptic weights. The regression problem can be solved using linear regression in matrix form:

(5) $$w^{T}=Y^{T}\cdot Z\cdot {\left(Z^{T}\cdot Z\right)}^{-1}$$

in which Z is a matrix of the hidden layer outputs for all data points, and Y is a vector containing all the target values.

### The PSO-NSFM Algorithm

As the synaptic weights can be trivially calculated using (5), the most cumbersome part of the training procedure in RBF networks involves calculation of the number and locations of the hidden kernel centers. Conventional training techniques like the *k*-means algorithm [34] postulate an arbitrary number of RBF kernels and then calculate their locations, the final selection being made through a trial-and-error procedure.

An alternative to this time-consuming approach was given by the fuzzy means algorithm (FM) [35], which has the ability to calculate in one step the number and locations of the RBF kernel centers and has found many successful applications in diverse fields like earthquake estimation [36], medical diagnosis [37], categorical data modelling [38], etc. In a recent publication [32], a variant of the FM algorithm, namely the PSO-NSFM algorithm, was proposed. The PSO-NSFM algorithm presents several remarkable advantages, including higher prediction accuracies in shorter computational times, accompanied by simpler network structures. What follows is an overview of the algorithm; the interested reader can refer to the original publication.

Like the original FM algorithm, the PSO-NSFM variant is also based on a fuzzy partition of the input space. However, in this case the partition is non-symmetric, which implies that a different number of fuzzy sets $A_{i,j}=\left\{a_{i,j},\delta a_{i}\right\}$ is used to partition each input variable, where $a_{i,j}$ is the kernel center element and $\delta a_{i}$ is half of the width of the respective fuzzy set. Combining N 1-D fuzzy sets, one can generate a multi-dimensional fuzzy subspace. These fuzzy subspaces form a grid, in which each node is a candidate to become an RBF kernel center. The main objective of the PSO-NSFM algorithm is to assemble the RBF network hidden layer by selecting only a small subset of the fuzzy subspaces. This selection is made based on a hyper-ellipse placed around each fuzzy subspace center, described by the following equation:

(6) $$\sum_{i=1}^{N}\left(\frac{{\left(a_{i,j_{i}}^{l}-u_{i}\left(k\right)\right)}^{2}}{N{\left(\delta a_{i}\right)}^{2}}\right)=1$$

The hyper-ellipse is used to mark the boundary between input vectors that receive non-zero or zero membership degrees to each particular fuzzy subspace. Having defined the membership function, the algorithm proceeds with finding the subset of all the fuzzy subspaces that assign a non-zero multi-dimensional degree to all input training vectors. Notice that within the FM algorithm context, the number of selected RBF kernel centers is bounded by the number of training data, although, depending on the distribution of input data, a smaller number of kernels is usually produced. The selection is accomplished using a non-iterative algorithm that requires only one pass of the input data, thus rendering the kernel center calculation procedure extremely fast, even in the presence of a large database of input examples. Taking advantage of the short computational times, a particle swarm optimization (PSO)-based engine is wrapped around the kernel center selection mechanism, designed to optimize the fuzzy partition. The result is an integrated framework for fully determining all the parameters of an RBF network.

## 3. Inverse Controller Design

A controller employing an inverse neural model is based on an approximation of the inverse system dynamics, i.e., a dynamical model able to predict the manipulated variable value that drives the system to the desired setpoint, taking account of its current state. Consider the following dynamic system:

(7) $$\dot{x}\left(t\right)=f\left(x\left(t\right),v\left(t\right)\right)$$

in which x is the state vector, and v is input to the system. Function f can be assumed to be nonlinear, without any loss of generality.

### 3.1. RBF-Based Inverse Controllers and BIBS Stability

Assuming that (a) all the state variables can be measured, and (b) the available training examples are sufficient, the PSO-NSFM algorithm can be applied to approximate a discrete inverse dynamic function, thus generating the following closed loop control law:

(8) v(k)=RBF(x(k),ω(k))

in which ω is the setpoint value and RBF stands for the nonlinear function corresponding to the RBF network response, calculated through (4). The discrete signal generated by (8) can be easily converted to continuous through a zero-order hold element (9), and consequently fed back to the system.

**Figure 2:**
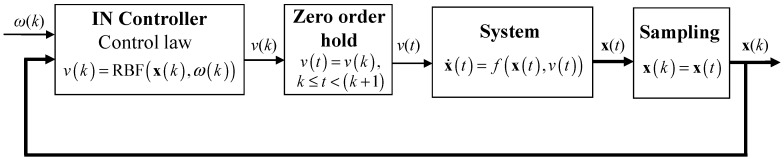
Closed loop with a simple RBF IN control scheme.

(9) v(t)=v(kT),kT≤t<(k+1)T

in which T is the sampling time. For the sake of simplicity, T is set equal to 1 in the following theoretical analysis. As can be observed, the trained RBF NN acts as a controller, by receiving as input the values of the current state vector and the setpoint and by producing as output the current manipulated variable value. Incorporation of the RBF inverse neural (IN) controller (8) in the closed loop is depicted in Figure 2.

As is shown below, the particular choice of control law guarantees that the closed loop system represented by (7)–(9) will be BIBS stable, if the open loop system (7) is BIBS stable.

**Lemma** **1.** *When the Gaussian basis function (2), and the PSO-NSFM algorithm are employed for producing the RBF-based inverse controller, the magnitude of the control law response* |v(k)| *produced by (8) is always upper bounded by* $\sum_{l=1}^{L}\left|w_{i}\right|$*.*

**Proof.**  As stated in the previous section, the application of the PSO-NSFM algorithm produces an RBF network with a finite number of basis functions L, which cannot exceed the number of training data K. Furthermore, the response of each Gaussian RBF is bounded from above and below, as follows:

(10) $$0\leq g\left(\mu_{l}\right)\leq 1,l=1,...,L$$

The control law response v(k) to any input vector $u\left(k\right)=\left[\begin{array}{cc} x\left(k\right) & \omega \left(k\right) \end{array}\right]$ is then given by:

(11) $$v\left(k\right)=\sum_{l=1}^{L}w_{l}g\left(\mu_{l}\left(u\left(k\right)\right)\right)$$

Lemma is proven by contradiction. Assume that the opposite is true, i.e., $\left|v\left(k\right)\right|>\sum_{l=1}^{L}\left|w_{i}\right|$. Then:

(12) $$\begin{array}{l} \left|\sum_{l=1}^{L}w_{l}g\left(\mu_{l}\left(u\left(k\right)\right)\right)\right|>\sum_{l=1}^{L}\left|w_{i}\right|\Rightarrow \\ \sum_{l=1}^{L}\left|w_{l}g\left(\mu_{l}\left(u\left(k\right)\right)\right)\right|\geq \left|\sum_{l=1}^{L}w_{l}g\left(\mu_{l}\left(u\left(k\right)\right)\right)\right|>\sum_{l=1}^{L}\left|w_{i}\right|\Rightarrow \\ \sum_{l=1}^{L}\left|w_{l}\right|\left|g\left(\mu_{l}\left(u\left(k\right)\right)\right)\right|>\sum_{l=1}^{L}\left|w_{i}\right|\Leftrightarrow \\ \sum_{l=1}^{L}\left|w_{l}\right|g\left(\mu_{l}\left(u\left(k\right)\right)\right)>\sum_{l=1}^{L}\left|w_{i}\right|\Leftrightarrow \\ \sum_{l=1}^{L}\left|w_{l}\right|\left(g\left(\mu_{l}\left(u\left(k\right)\right)\right)-1\right)>0 \end{array}$$

The last inequality requires $g\left(\mu_{l}\right)>1$ for at least one of the basis function responses, which leads to a contradiction with (10). ☐

**Theorem** **1.** The closed loop system represented by Equations (7)–(9) is BIBS stable, if the open loop system (7) is BIBS stable.

**Proof.**  Subject to the requirements imposed by the previous lemma, the control law response v(k), which is presented as input to (7) within the closed loop (7–9), is upper and lower bounded for every value of ω(k). Therefore, the input to system (7) is always bounded, and consequently all states are bounded, proving that the closed loop system (7) is BIBS stable. ☐

Notice that closed loop BIBS stability cannot be guaranteed for open loop BIBS stable systems when using different basis functions that are unbounded, e.g., the thin-plate-spline function [31].

### 3.2. Incorporating the AD Concept

All black-box techniques rely on the concept that the behavior of an unknown system can be modeled based solely on input-output data from it. After creating the black-box model by implementing a suitable learning algorithm on a training dataset, the model is utilized so as to provide predictions for new data points. However, as the only source of information relies on the available training data, it is expected that the more different the new data are compared to the data used in the training phase, the less reliable the model predictions will be. This phenomenon is called extrapolation, and it is known to affect all black-box-based techniques, including NNs. Due to the fact that RBF networks offer local approximation, they are particularly prone to poor performance when extrapolating.

Obviously, the inverse controller based on RBF networks described by (8) is also affected by extrapolation, a fact which can gravely degrade the controller performance. It must be noted that, even if special care is taken to collect training data that sufficiently cover the input space in terms of the state variables, this still leaves out the last element of the input vector, which is the current setpoint value. Thus, moving the system from the current state x(k) to the setpoint ω(k) within one discrete time step may not be feasible; such a situation could occur if the setpoint ω(k) is far from the current state of the system, while at the same time a relatively small sampling time is applied. In this case, extrapolation is inevitable, as the neural model is asked to produce a prediction without having been presented with any similar examples during its training phase.

The concept of AD is used in order to give an indication of whether a model performs extrapolation and to thus characterize the reliability of the model prediction [33]. An input vector u(k) is considered to fulfill the criterion of the applicability domain when the following expression is true:

(13) $$u\left(k\right)\cdot {\left(U^{T}\cdot U\right)}^{-1}\cdot u^{T}\left(k\right)\leq 3\frac{N+1}{K}$$

in which N is the number of input variables, K is the number of data in the training dataset, and U is a matrix which contains all of the input training data:

(14) $$U=\left[\begin{array}{cccc} u_{1}\left(1\right) & u_{2}\left(1\right) & \ldots & u_{N}\left(1\right)\\ u_{1}\left(2\right) & u_{2}\left(2\right) & \ldots & u_{N}\left(2\right)\\ \vdots & \vdots & \vdots & \vdots \\ u_{1}\left(K\right) & u_{2}\left(K\right) & \ldots & u_{N}\left(K\right) \end{array}\right]$$

To avoid the extrapolation phenomenon in the inverse RBF model predictions, the criterion described by the applicability domain notion is integrated in the process of designing the controller; as is explained later, this also provides a way for tuning the controller’s performance. The following equation, derived by (13) after substituting the input data vector to the RBF controller, defines the marginal condition for avoiding extrapolation:

(15) $$\left[\begin{array}{cc} x\left(k\right) & \omega \left(k\right) \end{array}\right]\cdot {\left(U^{T}\cdot U\right)}^{-1}\cdot {\left[\begin{array}{cc} x\left(k\right) & \omega \left(k\right) \end{array}\right]}^{T}=3\frac{N+1}{K}$$

Equation (15) is second order, as far as the current setpoint value ω(k) is concerned. The two solutions $\omega_{\min}\left(k\right)$ and $\omega_{\max}\left(k\right)$ define the maximum and minimum value of ω(k), which guarantee that no extrapolation occurs.

**Figure 3:**
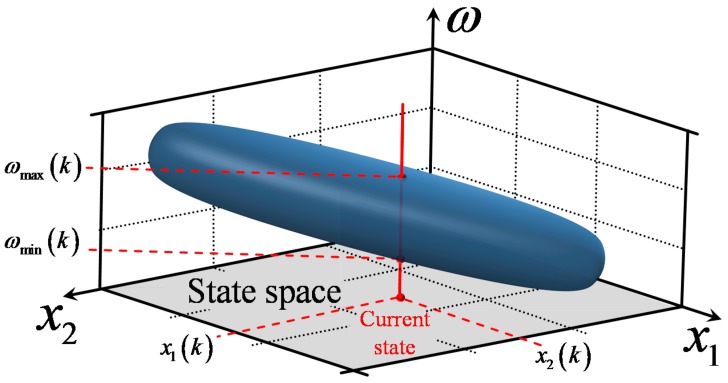
Calculating the bounds on the value of *ω*(*k*) that guarantee that extrapolation is avoided. The 3-D surface represents the AD of the RBF controller.

In order to visualize the application of AD to the inverse controller design, a system with two state variables $x_{1}$ and $x_{2}$ can be considered. Figure 3 depicts a 3-D graph of (15), in which the horizontal axes are the two state variables, while the vertical axis is the setpoint value ω(k). For each pair of state variables, (15) is solved and the resulting values $\omega_{\min}\left(k\right)$ and $\omega_{\max}\left(k\right)$ are plotted on the graph. The result is a 3-D surface, which represents the bounds of the RBF controller’s AD. It can be observed that for given values of $x_{1}$ and $x_{2}$, the two corresponding solutions $\omega_{\min}\left(k\right)$ and $\omega_{\max}\left(k\right)$ actually specify a line segment with length equal to $\omega_{\max}(k)-\omega_{\min}(k)$; when ω(k) receives values within this line segment, the input vector presented to the controller u(k) is guaranteed to be within the applicability domain; therefore, extrapolation is avoided.

The limits calculated by solving (15) express the marginal values for avoiding extrapolation. However, in many cases it is desirable to tighten those limits, e.g., in order to take into account inaccuracies that are present in the training data. To accomplish this, the length of the line segment defined by $\omega_{\min}\left(k\right)$ and $\omega_{\max}\left(k\right)$ can be divided by a narrowing parameter r, where r>1. Thus, stricter requirements are introduced to the AD, whose limits are given by:

(16) $$\begin{array}{l} {\omega^{\prime }}_{\min}\left(k\right)=\frac{\left(r+1\right)\omega_{\min}\left(k\right)+\left(r-1\right)\omega_{\max}\left(k\right)}{2r}\\ {\omega^{\prime }}_{\max}\left(k\right)=\frac{\left(r+1\right)\omega_{\max}\left(k\right)+\left(r-1\right)\omega_{\min}\left(k\right)}{2r} \end{array}$$

In those cases in which the setpoint ω(k) falls outside of the narrowed limits calculated by (16), it is replaced by the closest of these limits, according to the following equation, so as to keep the input vector inside the narrowed AD:

(17) $$\omega_{\mathrm{RE}}\left(k\right)=\left\{ \begin{array}{l} {\omega^{\prime }}_{\min}\left(k\right),if\;\omega \left(k\right)<{\omega^{\prime }}_{\min}\left(k\right)\\ {\omega^{\prime }}_{\max}\left(k\right),if\;\omega \left(k\right)>{\omega^{\prime }}_{\max}\left(k\right)\\ \omega \left(k\right),if\;\omega \left(k\right)\in \left[{\omega^{\prime }}_{\min}\left(k\right),{\omega^{\prime }}_{\max}\left(k\right)\right] \end{array}\right.$$

in which $\omega_{\mathrm{RE}}\left(k\right)$ is the requested setpoint value, replacing the original one in the control law, which now becomes:

(18) $$v\left(k\right)=\mathrm{RBF}\left(x\left(k\right),\omega_{\mathrm{RE}}\left(k\right)\right)$$

Utilization of the narrowing parameter r, as mentioned earlier, provides a means to tune the neural controller. Increasing the value of r results in more conservative control actions, as the requested setpoint value is closer to the current controlled variable value. On the other hand, smaller values of r make the control actions more aggressive, so that the extra distance to reach the requested setpoint is traversed.

### 3.3. Robustifying Term

The performance of neural controllers can be affected negatively by modeling errors due to disturbances, inadequacy of training data, etc. In order to take into account existing model-plant mismatches, a robustifying term is added to the model predictions with the purpose of error correction. More specifically, the error e(k) is calculated as the difference of the error-corrected setpoint of the preceding time step from the controlled variable value of the current time step:

(19) $$e\left(k\right)=\omega_{\mathrm{EC}}(k-1)-y\left(k\right)$$

in which y(k) is the current controlled variable value.

Assuming that the prior time step error remains constant throughout the next step, the requested setpoint is modified to compensate for the error; to accomplish this, the error term must be added to the requested setpoint $\omega_{\mathrm{RE}}\left(k\right)$ so as to calculate the error-corrected setpoint $\omega_{\mathrm{EC}}(k)$.

(20) $$\omega_{\mathrm{EC}}\left(k\right)=\omega_{\mathrm{RE}}\left(k\right)+\omega_{\mathrm{EC}}\left(k-1\right)-y\left(k\right)$$

It should be noted that the assumption of constant error for the next step is a typical approach that has also been used extensively in robust MPC [39]. Finally, (20) is substituted in (18) to produce a new control law:

(21) $$v\left(k\right)=\mathrm{RBF}\left(x\left(k\right),\omega_{\mathrm{EC}}\left(k\right)\right)$$

Notice that by narrowing the controller’s AD, which means that the tuning parameter r is increased, the allowed setpoint changes between two consecutive time steps become accordingly smaller. This ultimately results in smaller changes of the input vector component concerning the requested setpoint $\omega_{\mathrm{RE}}(k)$, a fact that strengthens the assumption of constant prediction error between two successive steps.

**Figure 4:**
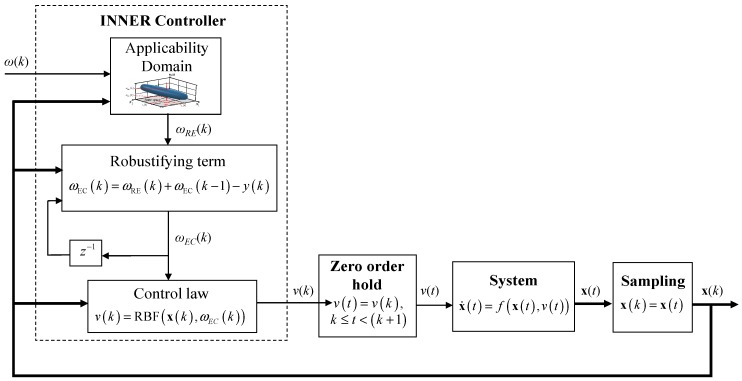
Closed loop with the RBF INNER control scheme, taking into account the applicability domain and the robustifying term.

The necessary modifications to the closed loop induced by adding the applicability domain and the robustifying terms are shown in Figure 4.

## 4. Case Studies

The resulting inverse neural non-extrapolating robustifying (INNER) controller is applied to the control of two different systems, namely, an experimental DC motor and a simulated nonlinear inverted pendulum. For comparison purposes, additional control schemes are tested, including a simple IN controller, a discrete PID for the case of the DC motor, and an analogue PID for the case of the inverted pendulum.

In both cases, each PID controller is tuned by linearizing each system using its respective state equations and then applying the internal model control (IMC) procedure. More specifically, the closed loop mean absolute error (MAE) criterion is minimized by using a 1st order filter. MAE is calculated as follows:

(22) $$\mathrm{MAE}=\frac{\sum_{k=1}^{K_{t}}\left|\omega \left(k\right)-y\left(k\right)\right|}{K_{t}}$$

in which $K_{t}$ is the number of simulation time steps. To be more specific, the IMC tuning parameter λ is optimized by trial and error, so as to achieve the lowest possible value for MAE. More details about the IMC procedure for PID tuning can be found in [40]. As far as the IN controller is concerned, no special tuning procedure is required, as this control scheme employs the exact same inverse neural model as the INNER control scheme. There are no parameters available for tuning and, thus, the performance of this controller depends solely on the quality and accuracy of the inverse model. 

### 4.1. Control of an Experimental DC Motor 

The objective of this case study is to control the rotational speed of an experimental DC motor. The experimental setup used in this work is the MS150 modular system [41], which was developed by Feedback Instruments Ltd. The system under control is a permanent magnet DC motor, which has a maximum rotational speed of about 4000 RPM in both directions when unloaded. The modular platform also consists of a power supply unit, a servo amplifier, a tachogenerator, and a magnetic brake, used to increase the system inertia. Control signal calculations are performed in real-time with a sampling rate of 100 samples per second, on a PC with an Intel Core 2 Quad processor at 2.67 GHz and 4 GBs of memory. The interfacing between the MS150 system and the PC is provided by the Feedback 33–301 analogue control interface, which includes a set of digital-to-analog converters (DACs) and analog-to-digital converters (ADCs), and the Advantech PCI1711 data acquisition card, which communicates directly with the Matlab data acquisition toolbox. All the controllers used in this study are implemented in the Matlab environment. The DC motor is armature-controlled and is described by the following state equations, derived using fundamental electrical and mechanical laws [42]:

(23) $$\begin{array}{l} \frac{di_{a}}{dt}=\frac{V_{a}-R_{a}i_{a}-K_{e}\omega_{r}}{L_{a}}\\ \frac{d\dot{\theta }}{dt}=\frac{K_{t}i_{a}-B_{L}\omega_{r}}{J} \end{array}$$

**Figure 5:**
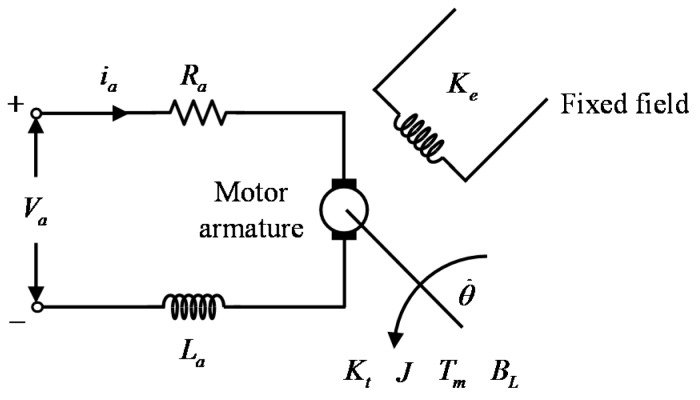
An armature-controlled DC motor.

The notation for the parameters appearing in (23) is given in Table 1, together with values for each of the DC motor parameters; a schematic of the DC motor is shown in Figure 5.

The inverse model used by the two neural controllers contains both electrical and mechanical variables and has the following form:

(24) $$V_{a}\left(k\right)=\mathrm{RBF}\left(i_{a}\left(k\right),\dot{\theta }\left(k\right),\dot{\theta }\left(k+1\right)\right)$$

In order to train the inverse RBF model, data are generated by drawing random changes from a uniform distribution, within ±15 V bounds, and applying them to the armature voltage $V_{a}$ every 0.5 s. The system sampling time is 0.01 s; thus, the armature voltage is kept steady for 50 samples, so that enough time is given for the system to reach steady state with each armature voltage value. The described configuration is used to collect 30,000 data points from the operation of the DC motor, which is divided into a 22,500-point training dataset and a 7500-point validation dataset. After data collection, the PSO-NSFM algorithm is applied, testing for partitions ranging from 4 to 40 fuzzy sets. Table 2 shows the results corresponding to the 5 top-performing networks found by PSO-NSFM, including the numbers of fuzzy sets and RBF kernel centers, the RMSE and *R*^2^ indices on the validation dataset, and the training time. Based on the values for RMSE and *R*^2^ achieved by the best network, it can be seen that the PSO-NSFM algorithm manages to develop a satisfactory inverse model of the system, especially taking into account that training is based on data from a real system, which inevitably includes noise. It should be noted that some of the remaining top-performing networks present a smaller number of RBF kernel centers, compared to the best network found. However, it was found experimentally that incorporating these models to the resulting control scheme produced inferior results in terms of MAE, and for this reason, the best performing network in terms of RMSE and *R*^2^ was selected.

In order to test the effectiveness of the proposed controller, a setpoint tracking problem, in which the objective is to follow a series of setpoint changes, is improvised in order to cover sufficiently the DC motor operating region.

**Figure 6:**
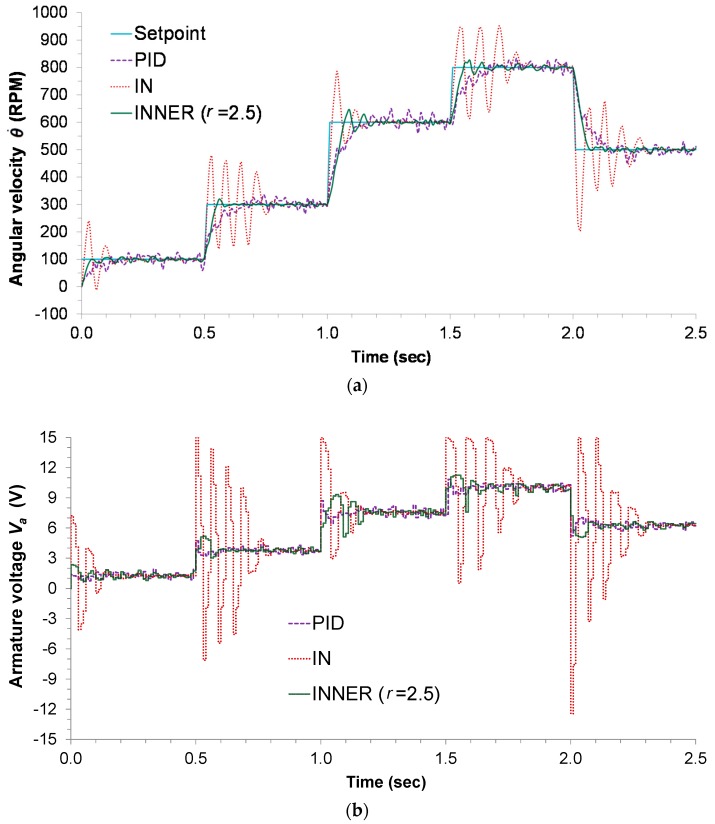
Armature-controlled experimental DC motor: (**a**) controller responses; (**b**) controller actions.

The only tuning parameter to be selected for the INNER controller is the narrowing parameter r. In order to optimize this parameter, different values of r are tested. A value of r=2.5 is selected, as it is found to produce the lowest MAE. Table 3 depicts the MAE values for the IN, INNER, and discrete PID controllers. Figure 6a depicts the respective responses, along with the setpoint changes, while the control actions can be seen in Figure 6b. The highly oscillatory response presented by the IN controller is the result of model inaccuracies, a problem that is alleviated by the INNER, which takes into account the AD; although this forces INNER to make smaller steps trying to reach the setpoint, it also aids in minimizing the overshoot and settling time. The oscillatory behavior of IN can also explain the significant difference in the MAE index between the two neural controllers, as INNER manages to avoid excessive oscillations by successfully employing the AD concept. The discrete PID controller also tracks all the setpoint changes, but is clearly inferior to INNER in terms of settling time, overshoot, and MAE. The higher MAE exhibited by the PID is attributed to the controller’s slow response and its inability to successfully counter any errors introduced by noise.

The successful application of the method on the DC motor indicates that it can handle issues that are associated with real world implementation, including the presence of noise, system-model mismatches, computational efficiency in real time applications, etc.

### 4.2. Control of a Simulated Inverted Pendulum

**Figure 7:**
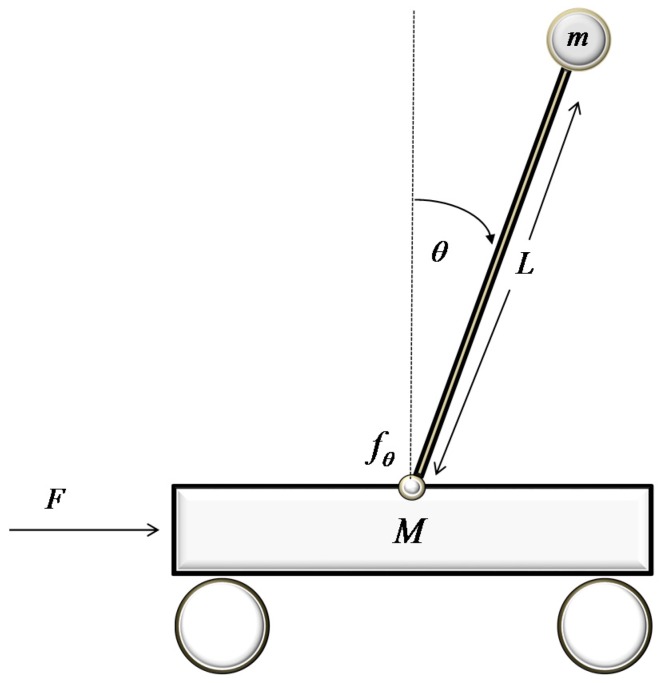
An inverted pendulum.

The second case study involves the implementation of the proposed controller in a problem closely related to the field of robotics, which has been identified as a standard benchmark for control, namely the control of an inverted pendulum. The inverted pendulum, as depicted in Figure 7, consists of a pole with a weight on one end, while the other end is attached on top of a small wagon. The wagon is connected to the pole through a pin that allows full range of motion in one level. Force F is applied on one side of the wagon in order to balance the pole on the vertical position, which constitutes an unstable equilibrium point. The inverted pendulum is described by the subsequent state equations, derived using fundamental physics laws:

(25) $$\begin{array}{l} \frac{dp}{dt}=v\\ \frac{dv}{dt}=\frac{-mg\sin\left(\theta \right)\cos\left(\theta \right)+mL{\dot{\theta }}^{2}\sin\left(\theta \right)+f_{\theta }m\dot{\theta }+F}{M+\left(1-\cos^{2}\left(\theta \right)\right)m}\\ \frac{d\theta }{dt}=\dot{\theta }\\ \frac{d\dot{\theta }}{dt}=\frac{\left(M+m\right)\left(g\sin\left(\theta \right)-f_{\theta }\dot{\theta }\right)-\left(Lm{\dot{\theta }}^{2}\sin\left(\dot{\theta }\right)+F\right)\cos\left(\theta \right)}{L\left(M+\left(1-\cos^{2}\left(\theta \right)\right)m\right)} \end{array}$$

The notation and values for the parameters appearing in (25) are given in Table 4. The manipulated variable in this case is the force F applied to the wagon, whereas the controlled variable is the angle θ. The inverse model formula shared by both neural controllers is given below:

(26) $$F\left(k\right)=\mathrm{RBF}\left(v\left(k\right),\theta \left(k\right),\dot{\theta }\left(k\right),\theta \left(k+1\right)\right)$$

The state Equations (25) are numerically solved in Matlab to produce a simulation of the inverted pendulum. The controllers are also implemented in the Matlab environment, and all simulations are run on a PC with an Intel i7 processor at 2.10 GHz and 8 GBs of memory.

Data generation and gathering to train the inverse RBF model is performed by randomly changing the force applied to the cart every 0.1 s, where the values are drawn from a Gaussian distribution ~N(0,6). After collecting a set of 40,000 data points, training and validation subsets are created, consisting of 30,000 and 10,000 data points, respectively. The next step is to apply PSO-NSFM for partitions employing 4 to 40 fuzzy sets; Table 2 presents details for the 5 best networks found by PSO-NSFM, ordered by the RMSE value. Once more, it can be seen that the produced models achieve high accuracy, as it can be seen by their RMSE and *R*^2^ values. The best network in terms of RMSE and *R*^2^ was found to produce better controller performance, as far as MAE is concerned, and thus it was used in the control problems that follow.

**Figure 8:**
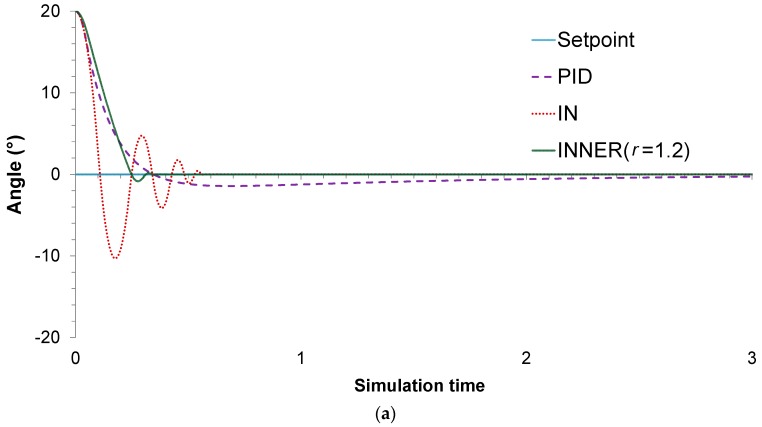

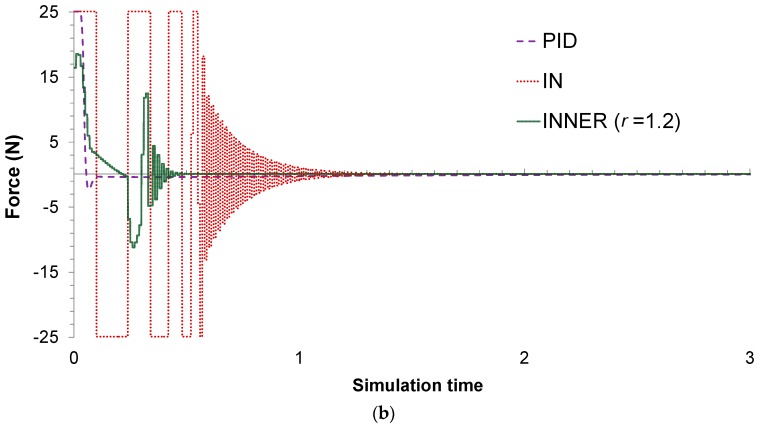
Inverted pendulum, (**a**) *M* = 1 kg: controller responses; (**b**) *M* = 1 kg: controller actions.

For this particular case study, two different control problems are improvised to assess the controllers’ performances. In the first one, the main objective is to stabilize the pendulum on the vertical position, when starting from an initial angle of 20°. A value of 1.2 is chosen for the r parameter, as it is found to produce the lowest MAE. The actual responses and control actions of the controllers are shown in Figure 8a,b, respectively, while the MAE values are summarized in Table 3. It can be seen that, compared to its rivals, INNER exhibits superior control performance in terms of the MAE values. The lower MAE values can be explained by the fact that INNER exhibits significantly faster stabilization time and lower oscillations, especially compared to IN. This can be understood by looking at the IN actions, which jump between the upper and lower saturation value until reaching an angle close to the setpoint, in contrast to INNER which provides less aggressive control actions. As far as the comparison with the analogue PID controller is concerned, the latter provides the most conservative control actions; however, INNER is much faster, exhibiting a significantly lower settling time. It must be mentioned that the PID starts with an advantage due to its analogue nature, in contrast with the neural controllers, which are allowed to change their actions only every 0.01 s. Despite this handicap, INNER manages to clearly outperform the analogue controller.

**Figure 9:**
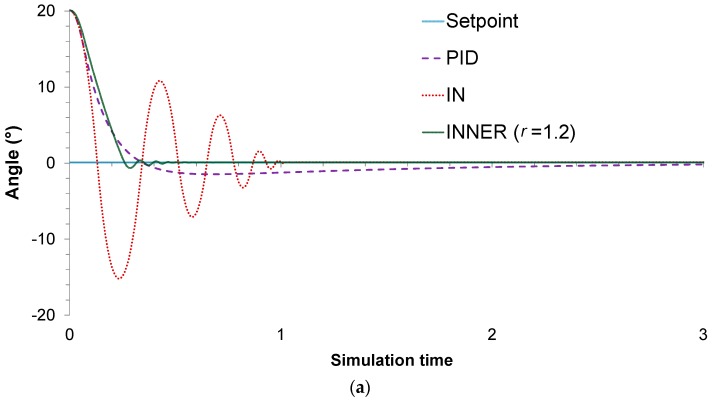

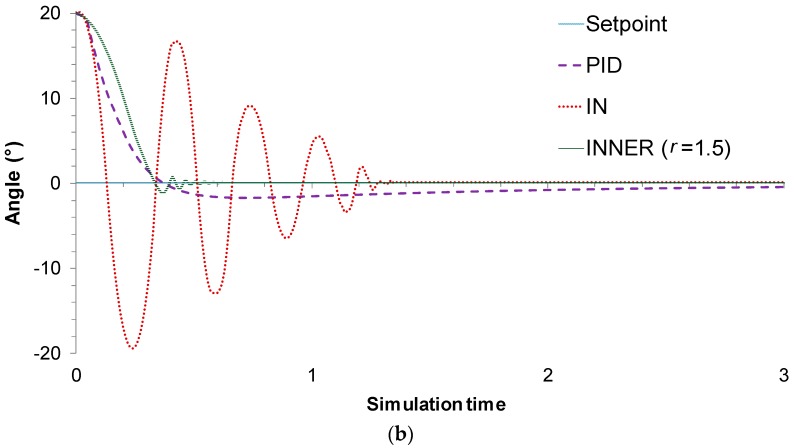
Inverted pendulum, (**a**) *M* = 1.4 kg: controller responses; (**b**) *M* = 2.0 kg: controller responses.

In the second control problem, the objective is once more to reach the vertical position when starting from an initial angle of 20°; however, in this case the mass of the wagon is altered to a value that is different compared to the value used during the data collection stage. This change allows us to assess the control schemes’ robustness. To enable a more detailed evaluation, two different values were used for wagon mass, namely *M* = 1.4 kg and *M* = 2 kg. The actual responses of the controllers for the two different values are depicted in Figure 9a,b, respectively, while the MAE values are given in Table 3. As seen in Figure 9a, all control schemes manage to reach the vertical position successfully for *M* = 1.4 kg; the proposed controller, though, proves to be superior compared to its rivals, as it exhibits a lower settling time, overshoot, and MAE compared to PID and IN.

The robustness of the proposed controlled is further validated in the case of *M* = 2 kg, in which it can be clearly seen that although the wagon’s mass has effectively doubled; the INNER is still able to surpass the rivaling control schemes regarding the settling time, overshoot, and MAE values. In order to achieve this, it was necessary to increase the value of the narrowing parameter *r* to 1.5. This change enables the INNER controller to compensate for the significant change in the wagon mass, albeit at the cost of a slightly slower response compared to lower values of *M*. The IN controller on the other hand, which lacks the robustifying capabilities of INNER, presents an excessive oscillation.

## 5. Conclusions

In this work, a new direct design methodology for generic neural controllers, which is able to control a nonlinear system given a sufficient volume of dynamic data collected during its operation, is presented. The control scheme is based on RBF networks with Gaussian basis functions; this choice makes certain that the control signal remains always bounded, and therefore BIBS stability for the closed loop is guaranteed when the open loop is BIBS stable. The described method uses an inverse dynamical RBF model of the system, which is able to combine inputs from different sources through a data fusion approach. The model is trained with the novel PSO-NSFM algorithm, which is found to improve both model accuracy and parsimony. Information drawn from the applicability domain of the model is used to break down the transition from the current system state to the requested setpoint to several smaller increments, with guaranteed feasibility. Moreover, a robustifying term for error correction is included, estimating the error due to model-plant mismatches, as well as unmeasured external disturbances and, thus, eliminating offset.

The resulting control scheme is tested through two control problems, namely, an experimental setup of a DC motor, as well as a simulated, highly nonlinear inverted pendulum. The proposed approach manages to successfully control both systems in all the cases that are tested, including setpoint tracking and unmeasured disturbance rejection, while it proves to be robust to model uncertainties. A comparison with two different controllers confirms the superiority of the proposed scheme. Future research plans include the exploitation of the generic nature of the proposed approach through application to the control of complex and uncertain nonlinear systems [43,44].

## Figures and Tables

**Table 1:** Notation and parameter values for the DC motor.

Parameter	Symbol	Description/Value
Rotor angular velocity	$\dot{\theta }$	State variable (RPM)
Armature current	*i_a_*	State variable (A)
Armature voltage	*V_a_*	Manipulated variable (V)
Armature resistance	*R_a_*	3.2 Ω
Armature inductance	*L_a_*	8.6 × 10^−3^ H
Back-EMF constant of motor	*K_e_*	100 × 10^−3^ V/rad/s
Torque constant of motor	*K_t_*	3.3 × 10^−3^ N∙m/A
Total moment of inertia	*J*	32 × 10^−6^ kg∙m^2^
Motor time constant	*T_m_*	250 × 10^−3^ s
Viscous friction coefficient of motor shaft	*B_L_*	128 × 10^−6^ N∙m∙s

**Table 2:** Specifications and statistics between the top performing trained RBF networks.

	Parameter	Fuzzy Partition	RBF Kernel Centers	RMSE Validation	*R*^2^ Validation	Training Time ^1^ (s)
System						
DC Motor		**[18 23 32]**	**242**	**9.7**	**0.93**	**398**
		[16 23 30]	210	9.8	0.93	
		[21 23 25]	227	9.8	0.92	
		[21 27 28]	270	10.0	0.90	
		[15 24 18]	199	10.3	0.89	
Inverted Pendulum		**[35 32 40 40]**	**189**	**0.48**	**0.98**	**912**
		[32 30 40 35]	173	0.50	0.97	
		[30 32 38 36]	179	0.50	0.97	
		[36 32 37 36]	180	0.52	0.96	
		[31 27 32 35]	151	0.56	0.91	

Bold numbers indicate the best model found for each system; ^1^ training was performed on a PC with an Intel i7 processor at 2.10 GHz and 8 GBs of memory.

**Table 3:** Values for Mean Absolute Error (MAE) in the two case studies.

Controller	MAE			
	DC Motor	Inverted Pendulum		
	Setpoint Tracking	Stabilization *M* = 1 kg	Stabilization *M* = 1.4 kg	Stabilization *M* = 2.0 kg
IN	0.595	0.315	0.676	0.8909
INNER	0.262	0.270	0.288	0.3201
PID	0.461	0.500	0.533	0.5581

**Table 4:** Notation and parameter values for the inverted pendulum.

Parameter	Symbol	Description/Value
Position of the wagon	*p*	State variable
Velocity of the wagon	*v*	State variable
Angle of the pendulum	*θ*	State variable
Angular velocity of the pendulum	$\dot{\theta }$	State variable
Force applied on the cart	*F*	Manipulated variable
Mass of the wagon	*M*	1 kg
Mass of the pendulum	*m*	0.5 kg
Gravitational constant	*g*	9.8 m/s
Length of the pendulum	*L*	0.3 m
Friction coefficient of the link	*f_θ_*	0.3 N/(m/s)
